# Measures of body habitus are associated with lung function in adults with cystic fibrosis: A population-based study^☆^

**DOI:** 10.1016/j.jcf.2012.08.008

**Published:** 2013-05

**Authors:** Doug L. Forrester, Alan J. Knox, Alan R. Smyth, Andrew W. Fogarty

**Affiliations:** a Nottingham Biomedical Research Unit, Department of Respiratory Medicine, City Hospital, Nottingham NG5 1PB, UK; b Nottingham Biomedical Research Unit, Division of Child Health, UK; c Nottingham Biomedical Research Unit, Division of Epidemiology and Public Health, UK

**Keywords:** Body mass index, Lung function, Muscle, Cystic fibrosis

## Abstract

**Background:**

Body habitus differences may explain some of the variation in lung function between individuals with cystic fibrosis (CF). We tested the hypothesis that measures of lean muscle mass and obesity are independently associated with lung function in CF.

**Methods:**

Cross-sectional study design using UK CF registry data from 2096 clinically stable adults.

**Results:**

Serum creatinine and BMI were positively and independently associated with FEV_1_ and FVC. One standard deviation increment in serum creatinine was associated with an FEV_1_ increase of 171 ml (95% confidence intervals CI: + 116 to + 227 ml) in males and 90 ml (95% CI: + 46 to + 133 ml) in females. Compared to the reference group of 20–24.9 kg/m^2^, those with a BMI < 20 kg/m^2^ had lower FEV_1_ with values of − 642 ml (95%CI: − 784 to − 500 ml) for males and − 468 ml (95%CI: − 564 to − 372 ml) for females.

**Conclusions:**

Prospective studies and controlled trials are required to ascertain if these associations have therapeutic potential in modifying disease progression.

## 1 Introduction

Cystic fibrosis (CF) is the most commonly inherited fatal disease in the Caucasian population. Characteristic features of the disease include recurrent pulmonary infections and malabsorption which contribute to accelerated loss of lung function and weight [2], and are associated with higher levels of mortality [3]. Hence, lung function and nutritional status are important clinical outcome measures that predict morbidity and mortality [4,5] and as a consequence are used to monitor the clinical condition of individuals with a diagnosis of CF. The most accessible measure of nutritional status is body mass index (BMI), and this is routinely recorded as part of the clinical data collection for CF.

There have been a number of smaller cross-sectional studies demonstrating that lung function as measured by Forced Expiratory Volume in one second (FEV_1_) is positively associated with BMI, consistent with the hypothesis that both are measures of disease severity [6–11]. Data from the CF Foundation Registry has consistently demonstrated that this inverse association between BMI and lung function is also present in population-based epidemiological data [12–14], and a study from the European Epidemiologic Registry of Cystic Fibrosis demonstrated lower function in those with decreased weight for height percentiles [15]. More recently, there have been a number of studies exploring the association between lean muscle mass (a different measure of body habitus), and measures of disease severity in CF [16–20]. Similarly to the earlier studies of BMI, the numbers studied are relatively small and it is unclear if these associations are present in larger, nationally representative populations.

Creatinine is released by skeletal muscle at a constant rate proportionate to total muscle mass and provides an epidemiological biomarker of total body skeletal muscle [21–23], with serum creatinine being strongly associated (r = 0.73) with fat-free mass [24]. We have recently demonstrated that serum creatinine is inversely associated with mortality in adult patients with cystic fibrosis [25]. Using the national UK database for CF, we tested the hypothesis that two measures of body habitus, serum creatinine and BMI, are positively associated with lung function in clinically stable adults with a diagnosis of CF. We quantified the relative contributions of these two exposures adjusting for potential confounding factors. Some of the results of these studies have previously been published in the form of an abstract.

## 2 Materials and methods

### 2.1 Study population and data collection

The study population consisted of individuals registered on the UK Cystic Fibrosis Registry aged 18 years or over who attended for an annual assessment visit in 2008 and were considered to be clinically stable by their physician. The registry is administered by the UK Cystic Fibrosis Trust and records information about the health and treatment of patients annually. Data are collected from 50 British CF care centres in a standardised anonymous fashion after obtaining patient consent. Each patient has an annual assessment visit when, among other investigations, lung function, weight, height and serum creatinine levels are measured and their clinical status is classified as stable or not. The main exposures of interest were serum creatinine, height and body mass index. The main outcome measure of interest was lung function as measured by forced expiratory volume in one second (FEV_1_) and forced vital capacity (FVC).

### 2.2 Statistical analysis

The data were visually examined for outlying values and any implausible values removed along with any values of creatinine greater than 300 μmol/L. The age was calculated for each individual on the 1st January 2008. The association between BMI and serum creatinine and FEV_1_ and FVC were explored using linear regression using quintiles of serum creatinine and BMI (coded as a categorical variable with the categories < 20 kg/m^2^, 20–24.9 kg/m^2^, > 25 kg/m^2^) adjusting for sex, age (coded as a categorical variable, < 20, 20–29, 30–39, 40 + years) and height as *a priori* confounding factors. The addition of height squared did not modify the size of associations by more than 10% and this was omitted from the final model. Likelihood ratio testing was used to test the linearity of these associations, and as the relation for serum creatinine and FEV_1_ was linear these data for this association was presented per standard deviation increment. The association between BMI and FEV_1_ was non-linear (p = 0.01) and hence this was presented as a categorical variable. The association between creatinine and FEV_1_ was modified by gender (LRT for interaction, p = 0.03), and the data were presented stratified for sex. Sensitivity analyses were used to explore the consistency of the associations observed, using different cut off points for creatinine of 150 μmol/L and 100 μmol/L to ensure that they were not being driven by extreme values. All analyses were performed using *STATA SE 11.0* (Stata Corporation, Texas) statistical software.

## 3 Results

**Fig. 1:**
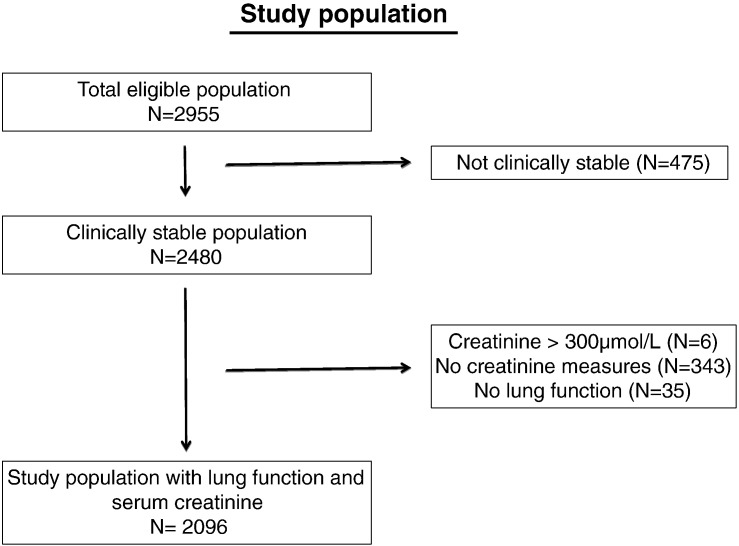
Flow diagram of study participants.

**Fig. 2:**
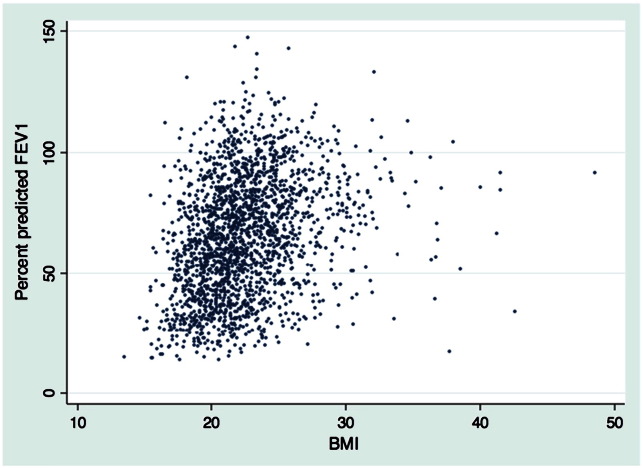
Association between lung function and body mass index.

2096 (56% male) patients attended for annual assessment in 2008 and provided complete data for analysis (Fig. 1). The clinical and physical status of those providing both FEV_1_ and serum creatinine data was not significantly different from those who did not (Table 1). For those who provided complete data, mean creatinine for males was 84.0 μmol/L (standard deviation[sd] 20.2 μmol/L) and for females was 68.6 μmol/L (sd 21.1 μmol/L). The basic data of the association between predicted FEV_1_ and BMI is presented in Fig. 2.

In the final multivariate model both serum creatinine and BMI were positively associated with FEV_1_ and FVC after mutual adjustment. After stratification for gender, a single standard deviation change in serum creatinine was associated with changes in FEV_1_ + 171 ml (95% confidence intervals[CI}: + 116 ml to + 227 ml) for males and + 90 ml (95%CI: + 46 ml to + 133 ml) in females respectively with similar changes seen in FVC (Table 2). The size of these associations was larger after using cuff off points of 100 μmol/L and 150 μmol/L as the highest level of creatinine values in the sensitivity analyses. Hence, the more conservative results are presented.

Modelled as a categorical variable using 20–24.9 kg/m^2^ as a reference group, BMI was strongly associated with lung function (Table 2). In males, those with a BMI of less than 20 kg/m^2^ had a FEV_1_ − 642 ml (95%CI: − 784 ml to − 500 ml) lower than the reference group, while those with a BMI greater or equal to 25 kg/m^2^ had a FEV_1_ + 364 ml (95%CI: + 227 ml to + 501 ml) greater than the reference group. Similar results were seen in for FVC and also in the analysis stratified for female sex.

## 4 Discussion

We have used national data from the United Kingdom Cystic Fibrosis Registry to demonstrate for the first time a positive linear association between an epidemiological measure of lean muscle mass (serum creatinine) and two measures of lung function, FEV_1_ and FVC. This is consistent with our hypothesis that total skeletal muscle mass is positively associated with lung function in this population. We have also confirmed and quantified the previously reported positive association between obesity as measured by BMI and lung function in a large epidemiological study population, having adjusted for the impact of muscle mass.

The strength of these data is the large, nationally representative nature of the UK national CF registry[26], that permits cross-sectional studies to be undertaken in the knowledge that almost the whole national patient population is covered by the database, thus increasing confidence in the conclusions that can be drawn from these data. One particular strength of the study was the ability to identify and exclude patients that were not considered to be clinically stable by the specialist clinic, minimising the risk of confounding by acute exacerbations that may reduce both lung and renal function. Another strength of our data was the ability to investigate the associations between obesity and lung function after adjustment for a measure of lean muscle mass, thus permitting calibration of the relative importance of these two measures on lung function for the first time. Finally, the linear nature of the associations observed and the consistency of our observations after sensitivity analyses using different cut off points for the ceiling level of serum creatinine, increases our confidence that these associations are not driven by extreme data points.

Our data have a number of limitations that require consideration. It is possible that some individuals may have had a subclinical infection at the time of their annual review that was not noted by the clinician involved. However, this would be expected to reduce the size of any associations observed rather than generate spuriously positive associations. In addition, the exclusion of patients who were not stable at the time of annual review suggest that our data cannot be generalised to this group of patients who may have more progressive disease. Also, we are unable to exclude the possibility that the associations that we are observing may be a consequence of confounding by other factors such as inflammation in early life [27]. Finally, the study population may contain some individuals who have received a lung transplantation and are now classified as clinically stable, although the absolute numbers of individuals compared to the total population will be small (approximately 40/year [28]) and unlikely to influence the associations observed.

Our use of serum creatinine as an epidemiological marker of lean muscle mass also requires critical consideration. Clinicians have been aware for years that muscle mass is positively associated with serum creatinine, and that caution is required when estimating renal function in bodybuilders [29,30] or the elderly [31], where extremes of muscle bulk may make the serum creatinine unreliable. A consequence of muscle turnover, creatinine is released at a consistent rate from skeletal muscle [32]. Data from a study of 24 men who provided paired measures of plasma creatinine and 24-hour urinary creatinine excretion [21], a validated measure of lean muscle mass [33], demonstrated that total plasma creatinine correlated strongly with urinary creatinine excretion with a correlation coefficient of 0.82, suggesting that blood creatinine may be used as an indirect measure of muscle mass. Similarly, a more recent study in healthy adults presented a strong correlation (r = 0.73) between serum creatinine and fat-free mass [24], and another study of children with leukaemia demonstrated a correlation co-efficient of 0.52 for serum creatinine concentration with lean body mass [23]. Animal studies in dogs suggest that plasma creatinine can predict muscle mass to within 3.9% of observed muscle mass [21]. As a consequence of these studies, we have recently used serum creatinine as an epidemiological measure of lean muscle mass in cystic fibrosis demonstrating lower mortality for individuals with higher levels of serum creatinine [25] while other researchers have used it for similar purposes in a study of 1017 individuals with diabetes [22]. We consider it likely that there will be a large amount of measurement error in the use of serum creatinine as a biomarker for muscle mass in large populations, which may result in a reduction in the size of association seen compared to the true association.

The use of creatinine as a biomarker of lean muscle mass in adults with cystic fibrosis in our dataset is relatively novel and is susceptible to the many endogenous and exogenous influences intrinsic to CF that may confound the associations observed. These will include renal impairment in patients with CF [34], often but not always as a consequence of exposure to multiple courses of aminoglycoside antibiotics or the presence of a diagnosis of diabetes. However, we would anticipate that those with impaired renal function would have more severe disease, and as a consequence expect that higher creatinine would be inversely associated with lung function, when in fact the converse is observed in our data. In addition, the associations were consistent across the sensitivity analyses that eliminated higher values of serum creatinine above 100 μmol/L, and so we are confident that these associations are not a consequence of high outlying values. However, it is possible that our observation that serum creatinine is positively associated with lung function may be attenuated by some patients with more severe disease having lower lung function and higher levels of creatinine. If this is the case, then the true association between serum creatinine and lung function would be higher than indicated by our data. Future studies of these associations would benefit from using measures of serum cystatin C in addition to serum creatinine, to adjust for mild levels of renal impairment.

There have been a number of small studies in both children and adults that have used DEXA scans to assess muscle bulk in individuals with cystic fibrosis. These have generally reported that patients with cystic fibrosis have less lean body mass than healthy controls [16,18,20,35–40]. In addition, it has been demonstrated that fat free mass is positively associated with lung function [9,17,18,20,38,41,42]. However, to our knowledge, this is the first study to demonstrate that reduced lean muscle mass as measured by serum creatinine is positively associated with lung function in a nationally representative population of adults with cystic fibrosis. This association is qualitatively similar to that reported by ourselves in a population-based study that used 24-hour urinary creatinine excretion to quantify lean muscle mass [43]. However, the size of association differed between the two populations, with a one standard deviation increase in serum creatinine in our data from individuals with cystic fibrosis being associated with an increase in FEV_1_ of 90 to 171 ml, while a one standard deviation increment of 24-hour urinary excretion was associated with a 45 ml increase in FEV_1_. This may be a consequence of the measurement error in the two different methods of quantifying lean muscle mass, or alternatively may reflect a difference between the two populations.

As our data are cross-sectional, we are unable to draw any conclusions regarding causality and can only speculate as to possible explanations for this association. Firstly, the observed associations may be a consequence of damaged lungs reducing exercise capacity and hence resulting in lower muscle bulk [24], and this is consistent with the observation that skeletal muscle strength is reduced during an acute pulmonary exacerbation of CF [44]. Secondly, capacity to exercise, itself a predictor of survival [45] may modify skeletal muscle mass and also may impact on lung function by reducing respiratory muscle strength [19,46]. Finally, the association may be confounded by disease severity, probably via a combination of malabsorption, pulmonary infections and systemic inflammation [47,48]. As the positive associations between both BMI and fat free mass with lung function are seen in children with an average age of 13 years, the processes that underpin this association may be present from an early life onwards [9].

Although the association between body mass index or similar epidemiological anthropometric measures and lung function in cystic fibrosis has been previously reported [6–9,15,20], the previous observational epidemiological studies of these associations did not adjust for potential confounding factors such lean muscle mass. The positive association between body mass index and lung function in cystic fibrosis is the opposite of that observed in healthy populations of adults where body mass index is inversely associated with lung function [49], and explaining this paradox may increase wider understanding on relationships between BMI and lung function generally. Our data are consistent with national data from the CF Foundation Registry which also suggests a positive association between FEV_1_ and BMI, that continues to increase beyond the value of 25 m^2^/kg [13]. Although BMI is a validated measure of adiposity in adults [50], it has not been validated in CF and hence may be a composite of other anthropometric components in this disease.

Untangling these potentially complex relations between body habitus and lung function will require longitudinal study designs, including observational studies and randomised controlled trials, ideally over an extended period of time. Such interventions should consider the impact of optimising lean muscle mass on physiological and quality of life related outcome measures and could include exercise based training programmes [51] and dietary supplementation with amino acids [52]. CF multidisciplinary teams typically include a specialist physiotherapist and specialist dietician and are ideally placed to provide a platform for physical and nutritional interventions based upon emerging supportive evidence.

In conclusion we demonstrate that serum creatinine, a biomarker for lower lean muscle mass, and BMI are independently positively associated with lung function in CF. Randomised controlled studies are required to identify interventions that target these outcome measures with a view to improving clinical outcomes in cystic fibrosis.

## Support statement

Doug Forrester is funded by Wellcome Trust Fellowship WT088614MA.

## Role of the funding source

The funding source had no role in study design, data analysis or decision to submit the manuscript for publication.

## Conflict of interest statement

The authors have no conflict of interest that could bias the data presented.

## Figures and Tables

**Table 1:** Description of study population of clinically stable adults with cystic fibrosis who provided annual assessment data in 2008.

	Provided baseline data on serum creatinine and FEV_1_	Did not provide data on serum creatinine and FEV_1_
Number	2096	384
Male, (%)	1165 (56)	218 (57)
Age, years (%)		
18–19	299 [14]	60 [16]
20–29	1051 [50]	181 [47]
30–39	469 [22]	88 [23]
40 +	277 [13]	55 [14]
Age, years, (sd)	28.9 (9.0)	29.4 (10.0)
FEV_1_, L, (sd)	2.36 (1.03)	2.42 (1.00) N = 323
Predicted FEV_1_, %, (sd)	65.1 (24.2) N = 2079	67.8 (25.2) N = 283
FVC, L, (sd)	3.53 (1.2)	3.54 (1.17) N = 323
Predicted FVC, %, (sd)	82.7 (21.4) N = 2079	84.3 (22.3) N = 283
BMI, kg/m^2^ (%)		
< 20	503 [24]	85 [26]
20 to 24.9	1177 (57)	182 (56)
≥ 25	390 [19] N = 2070	60 [18] N = 327
Height, m, (sd)	1.68 (0.1) N = 2079	1.68 (0.1) N = 335
Serum creatinine, μmol/L, (sd)	77.2 (22.0) N = 2096	71.5 (23.9) N = 35

FEV_1_ = Forced Expiratory Volume in one second. FVC = Forced Vital Capacity. BMI = Body Mass Index.

**Table 2:** A multivariate analysis of the association between measures of body habitus and lung function in adults with cystic fibrosis.

	Number of individuals	FEV_1_ (ml)	FVC (ml)
*Creatinine (per sex specific sd change)*			
Male	1153	+ 171 (+ 116 to + 227)	+ 99 (+ 41 to + 157)
Female	917 (p_interaction_ = 0.03)	+ 90 (+ 46 to + 133)	+ 64 (+ 19 to + 110)
			
			
*Body mass index (kg/m^2^)*			
Male			
< 20	228	− 642 (− 784 to − 500)	− 663 (− 810 to − 515)
20–24.9	670	0	0
≥ 25	255	+ 364 (+ 227 to + 501)	+ 272 (+ 130 to + 415)
Female			
< 20	275	− 468 (− 564 to − 372)	− 507 (− 609 to − 405)
20–24.9	507	0	0
≥ 25	135 (p_interaction_ = 0.09)	+ 237 (+ 113 to + 360)	+ 192 (+ 61 to + 323)

Mutually adjusted model includes for sex, age (categorical variable), body mass index (categorical variable) and height. sd = standard deviation. FEV_1_ = Forced Expiratory Volume in one second. FVC = Forced Vital Capacity.
