# Supplementary material for: Measures of body habitus are associated with lung function in adults with cystic fibrosis: A population-based study
Source: J Cyst Fibros. 2013 May;12(3):284–9. doi: 10.1016/j.jcf.2012.08.008 (PMC3655260; doi:10.1016/j.jcf.2012.08.008)
Supplement: Supplementary file 1 — Supplementary materials. [file mmc1.doc]

***The Journal of Cystic Fibrosis***

Review File

MEASURES OF BODY HABITUS ARE ASSOCIATED WITHLUNG FUNCTION IN ADULTS WITH

CYSTIC FIBROSIS: A POPULATION-BASED STUDY

Doug Forrester; Alan Smyth; Alan Knox; Andrew Fogarty

**Review timeline:**

Submission date: 17 May 2012

Editorial Decision: 06 Jul 2012

Revision received: 24 Jul 2012

Additional Correspondence:

Accepted:

**Transaction Report:**

(Note: With the exception of the correction of typographical or spelling errors that could be a source of ambiguity, letters and reports are not edited. The original formatting of letters and referee reports may not be reflected in this compilation.)

***1st Editorial Decision***

Reviewers have now commented on your paper. You will see that they are advising that you revise your manuscript. If you are prepared to undertake the work required, I would be pleased to reconsider my decision.

Finally, I would appreciate if you could submit your revised paper by the 5^th^ August, 2012

Yours sincerely

Editor-in-Chief

Journal of Cystic Fibrosis

**REFEREE REPORTS**

**Editor**: I tend to concur with the comments of Reviewer 1 and suggest that much more must be taken into account.  If creatinine is a surrogate marker for lean muscle mass in healthy controls, can the same be said for CF patients given the use of nephrotoxic medications and co-morbidities (e.g. diabetes).  I find the connection between nutrtional health and lung health to be extermenly important and agree that lean muscle mass may be an important factor, but I am worried that using the data contained within the registry is far too incomplete.

**Reviewer 1**: This manuscript uses the UK CF Registry to assess 2096 adults with CF in regards to FEV1, Cr and BMI.  The hypothesis is that BMI and Cr are associated with lung function. While the observation that FEV1 and BMI are associated is not a particularly novel one, it does confirm previous observations using a new database.  The association between Cr and FEV1 is an interesting one.

**Comments**

1.     The introduction and discussion are so concise that they feel cursory, and they need to be expanded to better reflect the current state of knowledge in the field.  Data have been presented from the U.S. CF registry on the BMI and FEV1 relationship.  These need to be discussed.  Likewise factors which influence Cr in CF can be discussed.

2.     While using categorical variables for the primary analysis of BMI-FEV1 makes sense, there is little additional detail provided for each group beyond n, gender, and mention of the relationship being non-linear.  Mean BMI for each group? FEV1 percent predicted? Discussion of extreme outliers?  A figure showing BMI vs FEV1 data with individual data points or a line through the mean FEV for each BMI would be helpful to better understand the relationship.

3.     The discussion of the relationship between CF and FEV1 also feels cursory.  There is little if any mention of two key influences on this relationship: use of aminoglycosides and diabetes.  The patients with most severe lung disease are going to have been exposed to repeated courses of nephrotoxic agents.  Older patients with likely lower lung function are going to be at a higher risk for diabetes.  Without some discussion of this or sensitivity analysis with attempt to control for these factors, the Cr relationship is not very rigorous.

4.     What were the age categories in the model?

5.     How were lung transplant patients treated

**Reviewer 2:**

The authors present an analysis of the UK CF registry that evaluates the relationship between lean muscle mass, as assessed by serum creatine measurement, body mass index, and pulmonary function.  The analysis is straightforward and statistical methods appear appropriate.

In the methods, the authors imply that patients are considered stable or unstable based on the opion of their physician.  In order to interpret this study accurately, it's important to understand if there is a general definition of stable (not having a pulmonary exacerbation) or if any non-stable finding (such as weight loss or new onset of diabetes) would be included.  Overall it would be helpful to know how many patients were excluded due to instability.  It is very unlikely that including such patients would change the main results, but it is important in assessing generalizability of findings.

The introduction and discussion neglect a few key points that are extremely important.  One is the relationship between poor nutrition and mortality. While this is undoubtedly known to readers of JCF it's worth reiterating (for example Sharma R, et al. Thorax. 2001;56:746-750).  I also thought it was fascinating that BMI was "neutral" in the range currently set as a goal by the US Cystic Fibrosis Foundation (22kg/m2 for women and 23 for men) and improved with a BMI of 25 or greater.  This deserves more emphasis in the discussion.  Finally, since markers of cardiopulmonary fitness such as VO2 max are independently associated with survival in CF, and since exercise increases lean muscle mass, some further discussion of exercise as a contributor to the findings (as opposed to interventions is warranted.

***1st Revision - authors' response***

I tend to concur with the comments of Reviewer 1 and suggest that much more must be taken into account. If creatinine is a surrogate marker for lean muscle mass in healthy controls, can the same be said for CF patients given the use of nephrotoxic medications and co-morbidities (e.g. diabetes). I find the connection between nutritional health and lung health to be extremely important and agree that lean muscle mass may be an important factor, but I am worried that using the data contained within the registry is far too incomplete.

***Authors’ response.***

We addressed most of these concerns in the specific responses to Reviewer 1. We have also added a flow diagram so that the representativeness/completeness of the data is more obvious. In our opinion, this is probably the most complete dataset that is available to test the hypotheses considered in this manuscript.

**Reviewer 1.**

This manuscript uses the UK CF Registry to assess 2096 adults with CF in regards to FEV1, Cr and BMI. The hypothesis is that BMI and Cr are associated with lung function. While the observation that FEV1 and BMI are associated is not a particularly novel one, it does confirm previous observations using a new database. The association between Cr and FEV1 is an interesting one.

***Comment 1.***

The introduction and discussion are so concise that they feel cursory, and they need to be expanded to better reflect the current state of knowledge in the field. Data have been presented from the U.S. CF registry on the BMI and FEV1 relationship. These need to be discussed. Likewise factors which influence Cr in CF can be discussed.

***Authors’ response.***

We have extensively expanded the introduction and discussion (text below).

*(Introduction) Cystic Fibrosis (CF) is the most commonly inherited fatal disease in the Caucasian population. Characteristic features of the disease include recurrent pulmonary infections and malabsorption which contribute to accelerated loss of lung function and weight (2), and are associated with higher levels of mortality (3). Hence, lung function and nutritional status are important clinical outcome measures that predict both morbidity and mortality (4, 5) and as a consequence are used to monitor the clinical condition of individuals with a diagnosis of CF. The most accessible measure of nutritional status is body mass index (BMI), and this is routinely recorded as part of the clinical data collection for CF. There have been a number of smaller cross-sectional studies demonstrating that lung function as measured by Forced Expiratory Volume in one second (FEV1) is positively associated with BMI, consistent with the hypothesis that both are measures of disease severity (4-9). Data from the CF Foundation Registry has consistently demonstrated that this inverse association between BMI and lung function is also present in population-based epidemiological data (10-12), and a study from the European Epidemiologic Registry of Cystic Fibrosis demonstrated lower function in those with decreased weight for height percentiles (13). More recently, there have been a number of studies exploring the association between lean muscle mass (a different measure of body habitus), and measures of disease severity in CF (14-18). Similarly to the earlier studies of BMI, the numbers studied are relatively small and it is unclear if these associations are present in larger, nationally representative populations.*

*(Discussion) The use of creatinine as a biomarker of lean muscle mass in adults with cystic fibrosis in our dataset is relatively novel and is susceptible to the many endogenous and exogenous influences intrinsic to CF that may confound the associations observed. These will include renal impairment in patients with CF (31), often but not always as a consequence of exposure to multiple courses of aminoglycoside antibiotics or the presence of a diagnosis of diabetes. However, we would anticipate that those with impaired renal function would have more severe disease, and as a consequence expect that higher creatinine would be inversely associated with lung function, when in fact the converse is observed in our data.*

*In addition, the associations were consistent across the sensitivity analyses that eliminated higher values of serum creatinine above 100μmol/L, and so we are confident that these associations are not a consequence of high outlying values. However, it is possible that our observation that serum creatinine is positively associated with lung function may be attenuated by some patients with more severe disease having lower lung function and higher levels of creatinine. If this is the case, then the true association between serum creatinine and lung function would be higher than indicated by our data. Future studies of these associations would benefit from using measures of serum cystatin C in addition to serum creatinine, to adjust for mild levels of renal impairment.*

***Comment 2.***

While using categorical variables for the primary analysis of BMI-FEV1 makes sense, there is little additional detail provided for each group beyond n, gender, and mention of the relationship being non-linear. Mean BMI for each group? FEV1 percent predicted? Discussion of extreme outliers? A figure showing BMI vs FEV1 data with individual data points or a line through the mean FEV for each BMI would be helpful to better understand the relationship.

***Authors’ response.***

We have added percent predicted FEV1 and FVC to the description of the study population and provided a scatter plot (below) of the association between percent predicted FEV1 and BMI so that the spread of the raw data can be better appreciated by the reader. This is probably better than the mean values which could be influenced by outlying values in the highest and lowest groups. The sensitivity analyses used cut-offs of 300, 150 and 100 mg/dL for the serum creatinine, and the associations seen were consistent, suggesting that extreme outliers did not drive these associations. The use of categorical variables for BMI resulted in any outlying values being pooled in one of three categories so that they could not drive any of the associations observed. Figure 2. Association between lung function and body mass index

***Comment 3.***

The discussion of the relationship between CF and FEV1 also feels cursory. There is little if any mention of two key influences on this relationship: use of aminoglycosides and diabetes. The patients with most severe lung disease are going to have been exposed to repeated courses of nephrotoxic agents. Older patients with likely lower lung function are going to be at a higher risk for diabetes. Without some discussion of this or sensitivity analysis with attempt to control for these factors, the Cr relationship is not very rigorous.

***Authors’ response.***

We have directly and extensively addressed these issues in the discussion. We used sensitivity analyses of a variety of cut-offs to serum creatinine (300, 150, 100) and the associations reported remained consistent, which suggests that this is a true association that is not a consequence of outlying values. *The use of creatinine as a biomarker of lean muscle mass in adults with cystic fibrosis in our dataset is relatively novel and is susceptible to the many endogenous and exogenous influences intrinsic to CF that may confound the associations observed. These will include renal impairment in patients with CF (31), often but not always as a consequence of exposure to multiple courses of aminoglycoside antibiotics or the presence of a diagnosis of diabetes. However, we would anticipate that those with impaired renal function would have more severe disease, and as a consequence expect that higher creatinine would be inversely associated with lung function, when in fact the converse is observed in our data.*

*In addition, the associations were consistent across the sensitivity analyses that eliminated higher values of serum creatinine above 100μmol/L, and so we are confident that these associations are not a consequence of high outlying values. Hence, it is possible that our observation that serum creatinine is positively associated with lung function may be attenuated by some patients with more severe disease having lower lung function and higher levels of creatinine. If this is the case, then the true association between lean muscle mass and lung function would be higher than indicated by our data. Future studies of these associations would benefit from using measures of serum cystatin C in addition to serum creatinine, to adjust for mild levels of renal impairment.*

***Comment 4.***

What were the age categories in the model?

***Authors’ response.***

This has been added to the statistical methods.

*The association between BMI and serum creatinine and FEV1 and FVC were explored using linear regression using quintiles of serum creatinine and BMI (coded as a categorical variable with the categories <20 Kg/m2, 20-24.9Kg/m2, >25Kg/m2) adjusting for sex, age (coded as a categorical variable, <20, 20-29, 30-39, 40+ years) and height as a priori confounding factors.*

***Comment 5.***

How were lung transplant patients treated?

***Authors’ response.***

As this was a population-based study of clinically stable patients, if lung transplant patients had been classified as clinically stable, then they would have been included in the national study population. We do not anticipate that this would have make a difference to the associations of interest, as the proportion of lung transplant patients in the total population of clinically stable adults will be small. However, we have modified the text to consider this point. *Finally, the study population may contain some individuals who have received a lung transplantation and are now classified as clinically stable, although the absolute numbers of individuals compared to the total population will be small (approximately 40/year (28)) and unlikely to influence the associations observed.*

**Reviewer 2.**

The authors present an analysis of the UK CF registry that evaluates the relationship between lean muscle mass, as assessed by serum creatinine measurement, body mass index, and pulmonary function. The analysis is straightforward and statistical methods appear appropriate.

***Comment 1.***

In the methods, the authors imply that patients are considered stable or unstable based on the opinion of their physician. In order to interpret this study accurately, it's important to understand if there is a general definition of stable (not having a pulmonary exacerbation) or if any non-stable finding (such as weight loss or new onset of diabetes) would be included. Overall it would be helpful to know how many patients were excluded due to instability. It is very unlikely that including such patients would change the main results, but it is important in assessing generalizability of findings.

***Authors’ response.***

The definition of clinically stable was made by the attending physician at the time of the yearly assessment and hence is a clinical definition. This is an inevitable consequence of using national databases. As the reviewer will know, there are many causes of potential clinical ‘instability’, most, but not all involving respiratory or nutritional problems. To inform the reader as to the generalizability of the data presented, we have added a flow diagram which gives a clearer idea of the data presented. Despite the exclusion of those who were clinically unstable and those who did not provide data, we still have data on 71% of the UK adult population who presented for annual assessment, which is a very impressive achievement (by the UK CF Registry).

**Figure 1. Flow diagram of study participants**


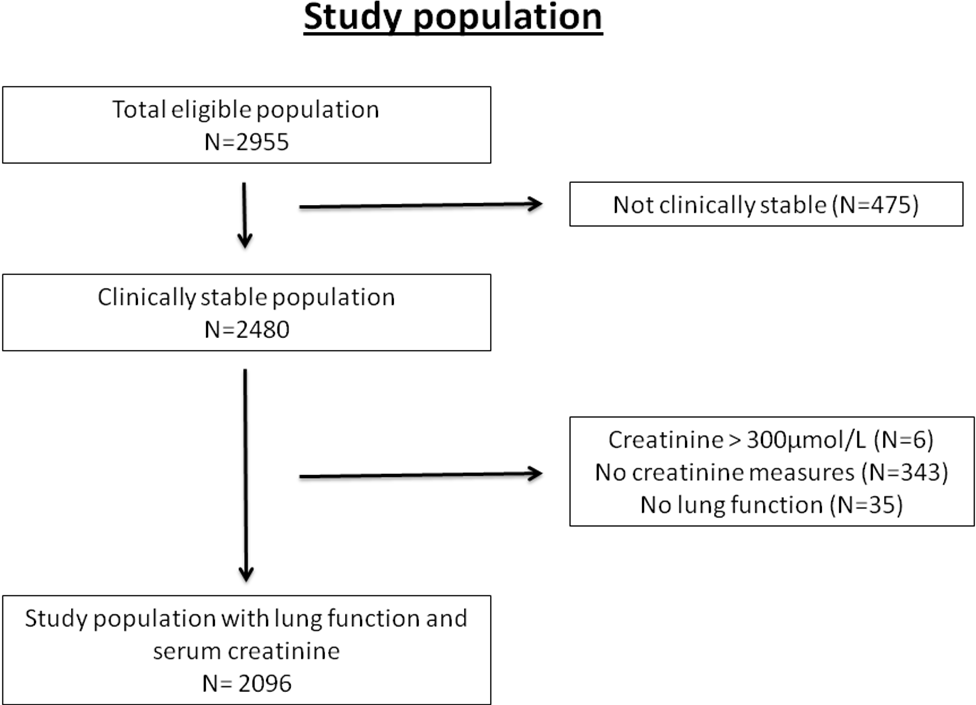


***Comment 2.***

The introduction and discussion neglect a few key points that are extremely important. One is the relationship between poor nutrition and mortality. While this is undoubtedly known to readers of

JCF it's worth reiterating (for example Sharma R, et al. Thorax. 2001;56:746-750). I also thought it was fascinating that BMI was "neutral" in the range currently set as a goal by the US Cystic Fibrosis Foundation (22kg/m2 for women and 23 for men) and improved with a BMI of 25 or greater. This deserves more emphasis in the discussion. Finally, since markers of cardiopulmonary fitness such as VO2 max are independently associated with survival in CF, and since exercise increases lean muscle mass, some further discussion of exercise as a contributor to the findings (as opposed to interventions is warranted).

***Authors’ response.***

We have added the following text to the introduction and discussion to address these points individually. (introduction) *Cystic Fibrosis (CF) is the most commonly inherited fatal disease in the Caucasian population. Characteristic features of the disease include recurrent pulmonary infections and malabsorption which contribute to accelerated loss of lung function and weight (2), and are associated with higher levels of mortality (3). Hence, lung function and nutritional status are important clinical outcome measures that predict morbidity and mortality (4, 5) and as a consequence are used to monitor the clinical condition of individuals with a diagnosis of CF. The most accessible measure of nutritional status is body mass index (BMI), and this is routinely recorded as part of the clinical data collection for CF.*  (discussion) *Our data are consistent with national data from the CF Foundation Registry which also suggests a positive association between FEV1 and BMI, that continues to increase beyond the value of 25m2/kg (13). discussion) Secondly, capacity to exercise, itself a predictor of survival (44) may modify skeletal muscle mass and also may impact on lung function by reducing respiratory muscle strength (19, 45).*

***2nd Additional Correspondence***

Thank you for submitting your revised manuscript for our consideration. We have now heard back from the referees who have reevaluated the study (see comments below), and I am pleased to inform you that both of them now consider the study suitable for publication. We shall therefore be happy to proceed with its acceptance and production.

Yours sincerely

Editor-in-Chief

Journal of Cystic Fibrosis
